# An Overview of Important Ethnomedicinal Herbs of *Phyllanthus* Species: Present Status and Future Prospects

**DOI:** 10.1155/2014/839172

**Published:** 2014-02-03

**Authors:** Bharti Sarin, Nidhi Verma, Juan Pedro Martín, Aparajita Mohanty

**Affiliations:** ^1^Department of Botany, Gargi College, University of Delhi, Siri Fort Road, New Delhi 110049, India; ^2^National Bureau of Plant Genetic Resources, IARI Campus, New Delhi 110012, India; ^3^Departamento de Biologia Vegetal, Escuela Tecnica Superior de Ingenieros Agronomos, Universidad Politecnica de Madrid, Ciudad Universitaria s/n, 28040 Madrid, Spain

## Abstract

The genus *Phyllanthus* consists of more than 1000 species, of which many are used as traditional medicines. The plant extracts have been used since ancient times, for treating hypertension, diabetes, hepatic, urinary, and sexual disorders, and other common ailments. Modern day scientific investigations have now confirmed pharmacognostic properties of *Phyllanthus *herbs. The phytochemicals attributing these medicinal properties have been identified in many of the *Phyllanthus* herbs. The morphologically similar herbs of *Phyllanthus* grow together and admixture of species during collection for manufacture of herbal medicines is quite common. Hence, along with pharmacognostic and phytochemical studies, appropriate protocols for correct identification of species are also important. As the use of these herbs as green medicines is becoming more popular, it is imperative to assess its genetic diversity and phylogenetic relatedness for future conservation strategies. This review is an attempt to present an overview of the existing studies on pharmacognostics, phytochemistry, species identification, and genetic diversity of *Phyllanthus* herbs and consequently (i) highlight areas where further research is needed and (ii) draw attention towards extending similar studies in underutilized but potentially important herbs such as *P. maderaspatensis*, *P. kozhikodianus*, *P*. *rheedii*, * P. scabrifolius, *and *P. rotundifolius.*

## 1. Introduction

The genus *Phyllanthus* (Phyllanthaceae) consists of approximately 1000 species, spread over the American, African, Australian, and Asian continents [1, 2]. All three major habits, that is, trees, shrubs, and herbs, are seen amongst the *Phyllanthus* species. Most of the herbs belonging to genus *Phyllanthus* have been shown to contain different combinations of secondary metabolites which render them with medicinal properties. The major class of bioactive compounds like alkaloids, flavonoids, lignans, phenols, tannins, and terpenes has been isolated from these herbs [3, 4].

Of a number of *Phyllanthus* herbs that are used all over the world as traditional herbal remedies, 12 important herbaceous species are discussed here. The species included are *P. ajmerianus* Rao and Choudhary, *P. amarus* Schum and Thonn, *P. debilis* Klein ex Wild, *P. fraternus* Webster, *P. kozhikodianus* Sivadasan and Manilal, *P. maderaspatensis* L., *P. rheedii* Wight, *P. rotundifolius* Klein ex Wild, *P. scabrifolius* Hook.f., *P. tenellus* Roxb, *P. urinaria* L., and *P. virgatus *G Forst. Of these 12 species, *P. ajmerianus* is reported only from India [5]. All of these herbs, except *P. ajmerianus*, *P. rotundifolius,* and *P*. *scabrifolius*, have been scientifically investigated and proven to be of pharmacological value. The ethnic tribes of India and other Asian countries have used the herbs of *Phyllanthus* species since ancient times, as traditional home remedies. The decoctions of various parts of the herbs are used for treating hepatic, urinary, and sexually transmitted diseases, diabetes, hypertension, cancer, and wounds. Taking cue from the ethnic medications and potential of herbal treatments, the modern society is now eager to resort to green medicines which are without adverse side effects. Many of the *Phyllanthus* herbs form an integral part of Ayurveda, an Indian system of medicine. Considering the importance and potential of these herbs, it is natural that most of the studies are directed towards the phytochemical analysis and pharmacognostics (references detailed in Tables 1 and 2). However, for appropriate utilization of the herbs for ethnopharmacological investigations and preparation of herbal medicines, the correct identification of *Phyllanthus* species is very important. Also, with the growing utilization of these herbs in pharmaceutical industries, the risk of loss of genetic diversity exists. There are comparatively a lesser number of reports focusing on molecular taxonomy for identification of species and interspecific/intraspecific genetic diversity studies.

Keeping this present scenario in view, 12 important *Phyllanthus* herbs (*P. ajmerianus, P. amarus, P. debilis, P. fraternus, P. kozhikodianus, P. maderaspatensis, P. rheedii, P. rotundifolius, P. scabrifolius*,* P. tenellus, P. urinaria, *and* P. virgatus*) are reviewed with the following objectives: (i) to assess and hence direct efforts towards further studies on phytochemistry and pharmacognostics of the important herbs, (ii) to focus on the need to initiate studies of underresearched but potentially important medicinal herbs, and (iii) to assess the existing studies on identification of species, genetic diversity, and phylogeny, which will have an impact on formulating conservation strategies in future. The detailed assessment of clinical studies pertaining to the *Phyllanthus* herbs has not been elaborated in the present review.

## 2. Pharmacognosy of *Phyllanthus *Herbs

Of the 12 *Phyllanthus* herb species, nine species (*P. amarus*, *P. debilis*, *P. fraternus*, *P. kozhikodianus*, *P. maderaspatensis*, *P. rheedii*, *P. tenellus*, *P. urinaria,* and *P. virgatus*) have been scientifically analyzed whereas the remaining three species (*P. ajmerianus*,* P. rotundifolius,* and *P. scabrifolius*) have not been investigated for their medicinal properties. The pharmacognostics of each of the nine herbs is discussed here.

### 2.1. *P. amarus*

This herb finds its use worldwide for treating problems of stomach, genitourinary system, liver, kidney, and spleen. It plays an important role in Ayurveda, an Indian system of medicine, and is used to treat jaundice, gastropathy, diarrhoea, dysentery, fevers, menorrhagia, scabies, genital infections, ulcers, and wounds [62]. Decoctions of whole plants are used for treating migraine, jaundice [63–65], gonorrhea and syphilis, skin disease, and malaria [66, 67]. Paste of leaves or its decoction [68–72] and juice of roots [73] are used for treating jaundice. Chronic dysentery, menstrual problems, anorexia, urinary tract infection, and diabetes are also treated by leaf extract taken orally [65, 69, 72, 74]. Extracts of the plant can prevent mutation of cells in the presence of chemical agents [75].

### 2.2. *P. debilis*

This herb shows antihepatotoxic [25] and anti-inflammatory [76] properties. Leaf juice is taken orally by the Kamar, Gond, and Halba tribes of Chattisgarh in India, for relief of problems related to sickle-cell anemia [77]. The aqueous extract of the plant shows antihyperglycemic property [78]. *P. debilis* has been shown to possess maximum antioxidant activity compared to *P. amarus*, *P. maderaspatensis*, *P. urinaria,* and *P. virgatus* [79].

### 2.3. *P. fraternus*

Traditionally, in India, the herb was used as a mild laxative, to expel worms and intestinal gas. The plant extracts are used for treating many types of biliary and urinary conditions like gall bladder, kidney stones, and bacterial infections such as cystitis, prostatitis, viral infections, hepatitis, flu, tuberculosis, liver diseases, anemia, veneral diseases, and urinary tract infections [80]. The antimicrobial property of *P. fraternus* has been reported by Chanda et al. [81]. The aqueous extract of the plant shows antioxidant property [82] and has protective effect against bromobenzene induced mitochondrial dysfunction [83]. Also the extract can reduce toxicity of drugs such as cisplatin and cyclophosphamide and therefore can be used to raise the therapeutic potential of anticancer drugs [84]. Ethanolic extract of the herb has antioxidant and anticoagulant property in experimental models [85]. According to Hukeri et al. [86], the flavonoids present in the herb show hypoglycemic effect in rats.

### 2.4. *P. kozhikodianus*

This herb provides protection to liver against chemical induced liver damage [87]. The herb was screened for hepatoprotective activity against liver damage induced by paracetamol in rats. Histological examination of liver confirmed hepatoprotective and antihepatotoxic properties [88].

### 2.5. *P. maderaspatensis*

Ethanolic extract of this herb demonstrated chemoprotective effect in modulating cisplatin-induced nephrotoxicity and genotoxicity, thus proving its antioxidative property [89]. This extract is also taken as a popular dietary supplement in the southern part of India. It has been experimented as an ameliorative for adriamycin-induced toxicity and oxidative stress in mice [90]. Whole plant extracts have shown antihepatotoxic, hepatoprotective, and choleretic activities [88, 91].

### 2.6. *P. rheedii*

The Muthuvan tribe of Kerala use all parts of this herb as a cure for liver diseases. The plant also shows hepatoprotective, antihyperglycemic, antihyperlipidemic, and antioxidant effects [92, 93].

### 2.7. *P. tenellus*

Extracts of fresh and dried plants have antiviral and antimicrobial activity [94, 95]. The callus extracts of this herb have potential analgesic properties against neurogenic and inflammatory pain [96]. Although this herb is beneficial for diabetes and treatment of hepatitis, urolithiasis, and bowel diseases, it induces depression, spasms, increased respiratory rate, and dyspepsia, as shown from experiments on mice [97].

### 2.8. *P. urinaria*

This herb has multiple uses with many pharmacognostic properties. Aqueous/methanolic extract of whole plant is used for treating cancer [98–100]. The ethanolic extract of this herb has anti-inflammatory and antioxidant activity [31]. The acetone extract of the plant has been found to inhibit herpes simplex virus infection [55, 101]. The plant parts have been successfully used in treating hypertension, jaundice, and diabetes [102]. Chloroform and methanolic extract have shown antibacterial activity against *Helicobacter pylori*, which causes peptic ulcers and gastric cancers [103]. Since *H. pylori* shows resistance to most antibiotics, this herb may be seriously studied for preparation of medicines against infections caused by this bacteria.

### 2.9. *P. virgatus*

In China, the extract of this herb is fed to children suffering from malnutrition due to worm infestation. This herb is used as an antiseptic and anti-inflammatory agent by the Gond tribe of India [104]. The plant extract shows high antioxidant property [105]. The lignin virgatusin is found in the plant parts and it inhibits growth of Gram-positive bacteria [61].

*Phyllanthus *herbs with potential ethnomedicinal properties that have not been scientifically analyzed are as follows.

### 2.10. *P. ajmerianus*

This herb is found in Ajmer in India, and its identity has been confirmed by Vishwanatha et al. [5]. This plant has not been assessed for medicinal properties.

### 2.11. *P. rotundifolius*

This species has been analysed along with other *Phyllanthus* herbs for hepatoprotective property and it was found that *P. urinaria* and *P. amarus* have comparatively higher potential than *P. rotundifolius* [106]. There are no other reports on its pharmacognostic properties.

### 2.12. *P. scabrifolius*

The occurrence of this herb was reported in Karnataka, India [107]. This is an endemic species and scientific evaluation for its economic uses is yet to be done. However, local people use plant's decoction for treating chronic gonorrhea and dysentery and as a diuretic. The paste of seeds is used on wounds. Leaf paste is used on scabies and elephantitis and roots find its use in treating jaundice.

## 3. Phytochemistry of *Phyllanthus* Herbs 

The major phytochemicals which have/may have a role in rendering the herbs with medicinal properties are listed in Table 1. Out of the 12 herbs reviewed, seven species (*P. amarus*, *P. debilis*, *P. fraternus*, *P. maderaspatensis*, *P. tenellus*, *P. urinaria,* and *P. virgatus*) have been reported to contain one or more classes of compounds such as lignans, flavonoids, tannins, and alkaloids.

Nahar et al. [4] have listed the various classes of phytochemicals found in *Phyllanthus *species. Of all the *Phyllanthus* herbs, the phytochemistry of *P. amarus* is well studied [62]. It has the maximum reports of pharmaceutically important compounds isolated from aqueous or organic solvent extracts. The lignans phyllanthin, hypophyllanthin, niranthin, nirtetralin, virgatusin, and heliobupthalmin lactone are common to *P. amarus*, *P. maderaspatensis*, *P. urinaria,* and *P. virgatus* [8]. However, according to Khatoon et al. [108], phyllanthin is absent in *P. maderaspatensis*. Also, Sharma et al. [106] have reported the absence of phyllanthin and hypophyllanthin from *P. maderaspatensis* and *P. urinaria*. In *P. fraternus*, phyllanthin is absent, according to the studies of Khatoon et al. [108], whereas Tripathi et al. [26] have reported that both phyllanthin and hypophyllanthin are present in *P. amarus* and *P. fraternus* but the concentration of these two lignans varies substantially in the two species. Presence of the lignan, phyltetralin, is common to *P. amarus*, *P. fraternus*, *P. maderaspatensis*, *P. virgatus,* and *P. urinaria *(Table 1). The lignan hinokinin has been isolated from *P. amarus*, *P. tenellus,* and *P. virgatus* [10]. Flavonoids such as rutin, quercitrin, quercetin, kaempferol, and astragalin are present in both *P. amarus* and *P. urinaria* [14, 39]. Presence of several ellagitannins such as geraniin, corilagin, and phyllanthusiins is also common to *P. amarus* as well as *P. urinaria* (Table 1).

To the best of our knowledge, there are no reports of isolation of any phytoconstituents from *P. ajmerianus*, *P. kozhikodianus*, *P. rheedii*, *P. rotundifolius,* and *P. scabrifolius*. These five herbs can be experimented for isolation and identification of pharmaceutically important phytochemicals.

### 3.1. Major Phytochemicals and Associated Pharmacological Activities

The phytochemicals with associated pharmacological activities of six *Phyllanthus* herbs (*P. amarus*, *P. debilis*, *P. fraternus*, *P. tenellus*, *P. urinaria,* and *P. virgatus*) are listed in Table 2.

The lignan phyllanthin renders hepatoprotective property to *P. amarus* [43]. However, *P. fraternus*, *P. maderaspatensis,* and *P. urinaria* do not contain phyllanthin [106, 108] but are hepatoprotective [106]. Therefore phyllanthin being the sole compound contributing to hepatoprotective property needs to be further investigated. Srirama et al. [109] also pointed out that phyllanthin and hypophyllanthin may not be the only compounds responsible for this property. According to Londhe et al. [52], hepatoprotective property of *P. amarus* is attributed to amariin and geraniin (which are ellagitannins), whereas phyllanthin and hypophyllanthin have been suggested to be anti-inflammatory and antiapoptotic [48].

Decalactone isolated from *P. debilis* is shown to possess antihepatotoxic ability [25]. The presence of this compound may be checked in other herb species which probably can throw light on potential of other *Phyllanthus* herbs as antihepatotoxic. Anticancer and/or antitumor properties have been related to the presence of phyllanthin, hypophyllanthin, niranthin, and polyphenols in *P. amarus* (Table 2). Of these three phytochemicals, niranthin is present in *P. fraternus*, *P. maderaspatensis*, *P. urinaria*, *P. virgatus* [8], and *P. tenellus* [10]. However, niranthin has not been investigated for anticancer potential in these herbs.

Antibacterial activity has been shown by phyllanthin and virgatusin in *P. amarus*. The lignin virgatusin from *P. virgatus* also shows antibacterial activity (Table 2). Virgatusin is present in *P. maderaspatensis *and *P. urinaria* as well (Table 1) and therefore the potential of this compound from these herbs can also be assessed for antibacterial activity. Antioxidant activity is shown by rutin, quercetin-3-O-glucoside (flavonoids), phyllanthin (lignan), amariin, repandusinic acid A, corilagin, phyllanthusiin A, B, C, geraniin (ellagitannins), methyl brevifolin (coumarin), methyl gallate, and trimethyl 1-3,4-dehydrochebulate (triterpenes) (see Table 2). Most of the compounds have been investigated in *P. amarus* for antioxidant property. Rutin and quercetin are found in *P. urinaria* but have been shown to exhibit antiviral property [31]. It may be worthwhile to investigate the role of rutin and quercetin for antioxidant property in *P. urinaria* and for antiviral property in *P. amarus*.

In separate studies, the antiviral property of *P. amarus* has been attributed to the compounds niranthin, nirtetralin, hinokinin, geraniin, and corilagin [10, 51]. The antiviral activity in *P. tenellus* and *P. virgatus* is also attributed to niranthin, nirtetralin, and hinokinin [10]. Geraniin is the common compound found in *P. amarus*, *P. urinaria,* and *P. virgatus*, which shows antiviral property in the three herbs [10, 55]. Anti-inflammatory activity in *P. urinaria* is attributed to the phytochemicals, phyltetralin, phyllanthin, quercetin, rutin, rhamnocitrin, and *β*-sitosterol [31, 59]. These compounds are found in other *Phyllanthus* herbs also (Table 1) and therefore the herbs can be assessed for anti-inflammatory property. Some of the flavonoids (rutin and quercetin-3-O-glucoside) and ellagitannins (geraniin, amariin, repandusinic acid, corilagin, and phyllanthusiin) in *P. amarus* have a role in radioprotective property (Table 2). Since most of these compounds are also present in *P. urinaria* (Table 1), the role of these phytochemicals in contributing radioprotective property in this herb can be investigated.

The alkaloid norsecurinine is associated with antifungal property of *P. amarus* and the compounds, *β*-sitosterol and *β*-amyrin, are associated with analgesic property of *P. urinaria* (Table 2). Two alkamides (E,E-2,4-octadienamide and E,Z-2,4-decadienamide) have been isolated from *P. fraternus* which contributes to the antiplasmodial property of the herb (Table 2). This is another pharmaceutically very relevant property that can be investigated for potential antimalaria drugs.

In the six herbs (i.e., *P. ajmerianus*, *P. kozhikodianus*, *P. maderaspatensis*, *P. rheedii*, *P. rotundifolius,* and *P. scabrifolius*), to the best of our knowledge, there are no studies correlating a phytochemical with its pharmaceutical property. The plant extracts of *P. kozhikodianus* and *P. rheedii* have been analysed for pharmacognostic properties and *P. kozhikodianus* is shown to be hepatoprotective [87, 88] and *P. rheedii* to have antihyperglycemic, antihyperlipidemic, and antioxidant effects [92, 93]. However, the compounds responsible for these pharmaceutical properties have not been identified. *P. ajmerianus*, *P. rotundifolius,* and *P. scabrifolius* have not been researched at all for either phytochemicals or pharmacological properties.

### 3.2. Phytochemicals with Multiple Pharmaceutical Properties

The lignan phyllanthin has been shown to possess maximum number of medicinal properties such as hepatoprotective, anticancer, antiamnestic, antiaging, antioxidant, and anti-inflammatory (Table 2). All these properties (except anti-inflammatory which is shown in *P. urinaria*) have been assessed in *P. amarus*. Another lignan, niranthin, is shown to have both antitumor and antiviral property. The flavonoids (rutin and quercetin-3-O-glucoside) and ellagitannins (geraniin, amariin, repandusinic acid, corilagin, and phyllanthusiin) have antioxidant as well as radioprotective property (Table 2).

## 4. Studies on Genetic Diversity, Species Identification, and Phylogeny

Genetic diversity studies, phylogenetics, identification of species, and characterization of germplasm are very important for appropriate utilization and conservation of plant genetic resources. The total number of genetic diversity studies on herbs of *Phyllanthus* is few, compared to pharmacognostic/pharmacological studies. The extent of genetic diversity has been investigated in *P. amarus*, *P. debilis,* and *P. virgatus* using RAPD (random amplified polymorphic DNA) and ISSR (Intersimple sequence repeats) markers, and an average polymorphism of 68.2% and 69.7%, respectively, was observed [110]. Genetic diversity analysis using RAPD markers, within *P. amarus* collected from different geographical locations in India, showed that the accessions from the southern part of India have high intrapopulation variation [111]. This variation may be attributed to the cross-pollination mechanisms in populations and also because they grow as weeds without much anthropogenic intervention [112].

Isozymes also have been used to assess the genetic variability in south Indian populations of *P. amarus* to identify superior genotypes for improving drug quality and for formulating strategies for *in situ *conservation and sustainable utilization [113]. Ravikant et al. [114] have also described southern India to be the genetic hot spot of *Phyllanthus* sp.

The ethnomedicinal uses and pharmacological activities among *P. amarus*, *P. fraternus*, *P. debilis,* and *P. urinaria* are varied but these plant species commonly grow together in the same open habitats and wastelands. In Bangladesh, China, India, Pakistan, and Thailand, *P. amarus*, *P. fraternus*, *P. debilis,* and *P. urinaria* grow together and lead to confusion in identification of these herbaceous species. Systematic studies on herbaceous *Phyllanthus *species, using morphological and anatomical parameters, could identify these *Phyllanthus* herbs [115]. Earlier, *P. amarus*, *P. fraternus,* and *P. debilis* were grouped under the single species named *P. niruri* and were later mentioned as species of “niruri complex.” Now, it is clarified that *P. niruri* is an American species and not at all found in India. Hence, the species identified as *P. niruri* is actually *P. amarus*, *P. fraternus,* and/or *P. debilis* [112, 116].

The use of molecular markers for identification of *Phyllanthus* species has proved to be a reliable tool. Species-specific SCAR (sequence characterized amplified regions) markers were developed for identification of *Phyllanthus* species (*P. amarus, P. fraternus, P. debilis,* and *P. urinaria*) used in dry leaf bulk trade [117, 118]. Bandyopadhyay and Raychaudhuri [119] compared RAPD, SCAR, and AFLP (amplified fragment length polymorphism) markers for identification of five *Phyllanthus* spp. and concluded that AFLP is a better polymorphic marker. Senapati et al. [120] identified species-specific diagnostic markers for ten *Phyllanthus* species, using intersimple sequence repeat-polymerase chain reaction (ISSR-PCR). Srirama et al. [121] assessed species admixtures in raw drug trade of *Phyllanthus* using DNA barcoding tools. They analyzed sequence variations of *psb*A-*trn*H region of the chloroplast to identify the *Phyllanthus* species present in the admixtures. AFLP profile along with morphological study could confirm the identification of *P. ajmerianus* Chaudhary and Rao in Ajmer, India [5]. The study also pointed to the distinct characteristic of the taxon and close relatedness to *P. kozhikodianus* and *P. rheedii*. PCR-RFLP approach of ITS (internal transcribed spacers) region has been successful in discriminating *P. amarus*, *P. debilis,* and *P. urinaria* [122].

Bandyopadhyay and Raychaudhuri [123] sequenced ITS regions of five species of *Phyllanthus*. The ITS sequences generated phylograms which aided in deducing affinities among *P. emblica*, *P. reticulatus*, *P. amarus*, *P. fraternus,* and *P. urinaria*. Phylogenetic relationships of 23 *Phyllanthus* species of Thailand have been analysed by sequencing ITS regions [122]. RAPD and ISSR markers have also been used to analyse phylogenetic relationship between twelve species of *Phyllanthus* [124].

## 5. Conclusions and Future Prospects

There are practically no studies on pharmacognostics and identification and/or isolation of pharmaceutically important compounds in *P. ajmerianus*, *P. rotundifolius,* and *P. scabrifolius*. Considering the medicinal properties of the *Phyllanthus* herbs, these species should be assessed for pharmacognostics and pharmacological properties. In case of *P. maderaspatensis, P. kozhikodianus,* and *P. rheedii*, although pharmacognostic properties are known, the compounds responsible for such properties have not been identified. Therefore these herbs can be experimented for isolation and identification of phytochemicals, based on existing knowledge of compounds observed in other *Phyllanthus* herbs and subsequently can be used for preparation of herbal medicines. Studies on pharmacognosy and pharmaceutical activity of phytochemicals published under the identity of *P. niruri* may be reevaluated especially the studies from India, as now it is confirmed that *P. niruri* is not found in India [112]. The studies, mentioning *P. niruri*, actually may be of *P. amarus*, *P. fraternus,* or *P. debilis*. Also, to our knowledge, there are either few or no studies on genetic diversity of most *Phyllanthus *herb species, some of which are endemic species with a few populations, for example, *P. scabrifolius* in India. Considering the growing popularity of ethnopharmacological value of *Phyllanthus* species and its use in herbal medicines, it is imperative to assess the genetic diversity of these species, which will have implications for formulating conservation strategies in future.

## Figures and Tables

**Table 1:** Major phytochemicals isolated from *Phyllanthus amarus*, *P. debilis*, *P. fraternus*, *P. maderaspatensis*, *P. tenellus*, *P. urinaria*, and *P. virgatus. *

Species	Class	Phytochemical	References
*P. amarus *	Lignans	Phyllanthin, hypophyllanthin	[6, 7]
		Niranthin, nirtetralin	[8, 9]
		Phyltetralin	[9]
		Heliobupthalmin lactone, virgatusin	[8]
		Isonirtetralin, lintetralin	
		Isolintetralin, demethylenedioxy-niranthin, 5-demethoxy-niranthin	[7]
		Hinokinin	[10]
		3-(3,4-Dimethoxy-benzyl)-4-(7-methoxy benzo[1,3] dioxol-5-yl-methyl)-dihydrofuran-2-one, 4-(3,4-dimethoxy-phenyl)-1-(7-methoxybenzol[1,3]dioxol-5-yl)-2,3-bis-methoxymethyl-butan-1-ol	[11]
	Flavonoids	Rutin	[12]
		Kaempferol, quercetin, quercitrin	[13]
		Astragalin, quercetin-3-O-glucopyranoside	[14]
		Quercetin-3-O-glucoside	[12]
	Tannins precursors	Gallic acid, gallocatechin	[15, 16]
		Ellagic acid	[17]
	Tannins	Corilagin	[14]
		Geraniin	[10]
		Amariin, furosin	[16]
		Repandusinic acid A	[18]
		Phyllanthusiin A, B, C, and D	[18]
		Geraniinic acid B, amariinic acid, amarulone, isocorilagin, elaeocarpusin	[15, 16]
	Alkaloids	Securinine	[19, 20]
		Dihydrosecurinine, tetrahydrosecurinine,	[20]
		Securinol, phyllanthin, allo-securine	[20]
		Norsecurinine	[21]
		Epibubbialine, isobubbialine	[22]
		Phenazine and phenazine derivatives	[15]
	Triterpenes	Ursolic acid, oleanolic acid	[23]
		2Z,6Z,10Z,14E,18E,22E-Farnesyl farnesol	[7]
		Lupeol, phyllanthenol, phyllanthenone	[14]
	Volatile oil	Linalool, phytol	[24]

*P. debilis *	Oxirano-furanocoumarin	Decalactone	[25]

*P. fraternus *	Lignans	Phyllanthin, hypophyllanthin	[26, 27]
		Niranthin, nirtetralin, phyltetralin	
	Alkamides	E,E-2,4-Octadienamide, E,Z2,4-decadienamide	[28]

*P. maderaspatensis *	Lignans	Phyllanthin, hypophyllanthin, niranthin, phyltetralin, nirtetralin, Heliobupthalmin lactone, virgatusin	[8]
	Lipids	Linoleic acid, linolenic acid, myristic acid, oleic acid, palmitic acid, stearic acid	[29]

*P. tenellus *	Lignans	Niranthin, nirtetralin, hinokinin	[10]
	Tannins	Pinocembrin-7-O-[4′′,6′′-(S)-hexahydroxydiphenoyl]-*β*-D-glucose, pinocembrin-7-O-[3′′-O-galloyl-4′′,6′′-(S)-hexahydroxydiphenoyl]-*β*-D-glucose	[30]

*P. urinaria *	Lignans	Phyllanthin, hypophyllanthin, niranthin, nirtetralin, virgatusin, heliobupthalmin lactone	[8]
		Phyltetralin	[31]
		5-Demethoxyniranthin	[32]
		Lintetralin, urinatetralin, urinaligran, dextrobursehernin	[33]
	Ellagitannin	Geraniin	[34]
		Ellagic acid	[35–37]
		Corilagin	[36, 38]
		Phyllanthusiin U	[36]
		Gallic acid	[35]
	Flavonoid	Quercitrin, rutin, astragalin, quercetin, isoquercitrin, kaempferol	[39]
		Rhamnocitrin	[31]
	Acid	Hexacosanoic acid	[40]
	Alkanol	Triacontanol	[40]
	Phytallate	Phyllester	[40]
	Sterol	*β*-Sitosterol, daucosterol	[35]
	Triterpenes	*β*-Amyrin, lupeol acetate	[31, 40]
		Methyl gallate, trimethyl 1-3,4 dehydrochebulate	
	Coumarin	Methyl brevifolin carboxylate	[31]

*P. virgatus *	Lignans	Phyllanthin, hypophyllanthin, niranthin, nirtetralin, heliobupthalmin lactone, virgatusin	[8]
		Hinokinin, isolintetralin, phyltetralin, (+)-8-3,4-methylenedioxybenzyl-8′-(3′,4′-dimethoxybenzyl)-butyrolactone	[41]
	Tannins	Virgatyne, virganin, norlignan	[42]
	Ellagitannins	Geraniin	[10]
	Flavonoid sulfonates	Galanin-8-sulfonate, galanin-3-O-beta-D-glucoside-8-sulfonate, Kaempferol-8-sulfonate	[42]
	Acid	Indole-3-carboxylic acid	[41]

**Table 2:** Pharmacological effect of major phytochemicals in *Phyllanthus amarus*, *P. debilis*, *P. fraternus*, *P. tenellus*, *P. urinaria*, and *P. virgatus*.

Species	Class	Phytoconstituent	Pharmacological effect	Reference
*P. amarus *	Lignan	Phyllanthin	Hepatoprotective	[43]
			Anticancer, antitumour	[44]
			Antileukemia	[45]
			Antibacterial	[46]
			Antiamnestic	[47]
			Antiaging
			Antioxidant	[6]
			Anti-inflammatory, antiapoptotic	[48]
		Hypophyllanthin	Antitumor, anticancer	[44]
		Niranthin	Antitumor	[20]
			Antiviral	[10]
			Anti-inflammatory	[9]
		Phyltetralin	Anti-inflammatory	[9]
		Nirtetralin	Anti-inflammatory	[9]
			Antiviral	[10]
			Reverses multidrug resistance	[49, 50]
		Hinokinin	Antiviral	[10]
	Flavonoid	Rutin	Radioprotective	[12]
			Antioxidant	[18]
		Quercetin-3-O-glucoside	Antioxidant	[18]
	Tannin	Geraniin	Antiviral	[10, 51]
			Radioprotective	[12]
			Hepatoprotective	[52]
		Amariin	Antioxidant	[18]
			Radioprotective	[12]
			Hepatoprotective	[52]
		Repandusinic acid A	Antioxidant	[18]
			Radioprotective	[12]
		Corilagin	Antioxidant	[18]
			Radioprotective	[12]
			Antiviral	[51]
		Phyllanthusiin A, B, C, D	Antioxidant	[18]
			radioprotective	[12]
	Alkaloid	Norsecurinine	Antifungal	[21]
	Volatile oil	Linalool, phytol	Antimicrobial	[24, 53]
	Polyphenol		Anticancer	[54]

*P. fraternus *	Alkamide	E,E-2,4-Octadienamide	Antiplasmodial	[28]
		E,Z-2,4-Decadienamide	Antiplasmodial	[28]

*P. debilis *	Oxirano-furanocoumarin	Debelalactone	Antihepatotoxic	[25]

*P. tenellus *	Lignan	Niranthin	Antiviral	[10]
		Nirtetralin	Antiviral	[10]
		Hinokinin	Antiviral	[10]

*P. urinaria *	Lignan	Phyltetralin	Anti-inflammatory	[31]
		Phyllanthin	Anti-inflammatory	[31]
	Flavonoid	Quercetin	Anti-inflammatory	[31]
		Rutin	Anti-inflammatory	[31]
		Rhamnocitrin	Anti-inflammatory	[31]
	Coumarin	Methylbrevifolin carboxylate	Antioxidant	[31]
	Ellagitannin	Geraniin	Antiviral	[55]
			Antioxidant	[56]
		Ellagic acid	Antiangiogenic	[57]
			Antiviral	[37]
	Sterol	*β*-Sitosterol	Analgesic	[58]
			Anti-inflammatory	[59]
	Triterpene	*β*-Amyrin	Analgesic	[60]
		Methyl gallate	Antioxidant	[31]
		Trimethyl 1-3,4 dehydrochebulate	Antioxidant	[31]

*P. virgatus *	Lignan	Niranthin	Antiviral	[10]
		Nirtetralin	Antiviral	[10]
		Hinokinin	Antiviral	[10]
		Virgatusin	Antibacterial	[61]
	Ellagitannin	Geraniin	Antiviral	[10]
